# RACIAL DISPARITIES IN NURSING HOME RESIDENTS: QUALITY OF LIFE: DOES THE GAP PERSIST OVER TIME?

**DOI:** 10.1093/geroni/igz038.2870

**Published:** 2019-11-08

**Authors:** Tetyana P Shippee, Weiwen Ng, John Bowblis, Yinfei Duan, Odichinma Akosionu, Mark Woodhouse

**Affiliations:** 1 University of Minnesota, Minneapolis, Minnesota, United States; 2 Dr, Minneapolis, Minnesota, United States; 3 University of Miami Ohio, Oxford, Ohio, United States

## Abstract

The proportion of racial/ethnic minorities in nursing homes (NHs) has increased steadily in recent years. This study longitudinally examines minority NH residents’ quality of life (QoL); a key measure of overall well-being. We used unique data from Minnesota annual QoL interviews (2011-2015), merged with resident and facility characteristics to model QoL. Mixed models with various resident and facility level controls, facility random effects, and both fixed and random effects for year were fit to estimate the effect of being a minority and living in a high-proportion minority facility on QoL. While white residents’ unadjusted QoL scores remained stable over time, scores for minority residents declined. In full models, white residents in low-minority facilities consistently had the highest QoL scores while minority residents in high-minority facilities had the lowest scores. More policy attention is needed to address these persistent and possibly widening racial disparities, with targeted attention needed for high-minority facilities.

